# Supplementary material for: The Baikal subtype of tick-borne encephalitis virus is evident of recombination between Siberian and Far-Eastern subtypes
Source: PLoS Negl Trop Dis. 2023 Mar 27;17(3):e0011141. doi: 10.1371/journal.pntd.0011141 (PMC10079218; doi:10.1371/journal.pntd.0011141)
Supplement: S1 Table — (PDF) [file pntd.0011141.s001.pdf]

| Strain ID | Sourse of isolation | Location of collection | Date of collection | I passage | II passage | III passage | IV passage | V passage | VI passage | VII passage |
|---|---|---|---|---|---|---|---|---|---|---|
| MT708809 | Ixodes persulcatus | Republic of Buryatia | 10.08.90 | SM | SM | SM | SM | SM | PEK* | |
| MT708810 | Ixodes persulcatus | Republic of Buryatia | 10.08.90 | SM | SM | SM | SM | PEK* | PEK | PEK* |
| MT708811 | Myodes rufocanus | Republic of Buryatia | 30.10.84 | SM | SM | SM | SM | PEK* | | |
| MT708812 | Myodes rufocanus | Republic of Buryatia | 28.10.84 | SM | SM | SM | SM | PEK | PEK* | |

**S1 Table: Isolation and cultivation of the studied variants.** Passages were done in suckling mouse (SM) brain tissue and pig embryo kidney (PEK) cell line; asterisk (*) marks sequenced RNA samples.
