# Supplementary material for: The Baikal subtype of tick-borne encephalitis virus is evident of recombination between Siberian and Far-Eastern subtypes
Source: PLoS Negl Trop Dis. 2023 Mar 27;17(3):e0011141. doi: 10.1371/journal.pntd.0011141 (PMC10079218; doi:10.1371/journal.pntd.0011141)
Supplement: S2 Table — (PDF) [file pntd.0011141.s002.pdf]

Name	Sequence (5’- 3’)
TBE83s	AGTCGTGAACGTGTTGAGAAAAAGA
TBE595a	CCATGTCMGTRGCCAGIATCACACAGGT
TBE538s	GCGGCAACYCAGGTGCGYGTGG
TBE1130a	GGGTTCTCCTGRTARATGGAGTCAA
TBEE1	TCACGGTGCACACATCTGGAAAA
TBE-6	CTTGGTTCCCTCAATGTGYGCCACAGGAA
RTBE1-4	GTTGACYTKGCYCAGACYGTCAT
TBEE2	CATCAGCTCCCACTCCGAGTGTCAT
TBE2377s	CTGAACATGAGAAACCCTACAATGTCCA
TBE2849a	GCCTCGGGGAYRCTCCAGATCA
TBE2708s	AGGCAAATCTCACAGTGGTGGTGGA
TBE3253a	CCTGTTCTGAATAGCCAGGTATCCTGTT
TBE3156s	CGACAATGCTGASGTSGTGGA
TBE3781a	ACCCTCARCTCAAACACRGCCTG
TBE3706s	GGGATCACATTCCAYCTYGAGC
TBE4284a	CTCCTGRGARGTGTGICGCATCA
TBE4162s	AGACGRTCWTTCAGTGAACCACT
TBE4677a	TCCTGTAGACACCRTCCYTAACCTC
TBE4546s	CTTGGWGTGATGGGAYTGTGGA
TBE5223a	TCTGTGGGTCTTCCCIGAGCCT
TBE5074s	CCAATGAGACYTAYGTCAGCAGCA
TBE5685a	CCCTCATACTCRGTGATCCAGT
TBE5633s	CCTGAKGGRGAGTGGCGTGA
TBE6164a	GCCACCTCYGGCATCTTGTCCT
TBE6096s	CGCAGATACTTCTTGACAAC
TBE6645a	CCCAGCACCATCTCHACCA
TBE6565s	CCTGGCAGCMGGGCAATGA
TBE7130a	CCAGWGGCCACRGCGCTGTTGAC
TBE7060s	TTCACWCCTTACATCATCCACCA
TBE7612a	CCCAIAGGCTRCCCCTGACCA
TBE7568s	GACACITTGTGGACGATGCC
TBE8115a	GCTYTCTCCRATGTCACACA
TBE8069s	GGGAATGGACGTGTTCAGCAT
TBE8681a	ACATCYTCCCGIGCGTTCCA
TBE8617s	GCGGCGTCWCTGATYAATGG
TBE9118a	CAAAGCGACTCCCCAGCCACA
FU1*	TACAACATGATGGGAAAGAGAGAGAA
TBE9505a	GGGTGTTGARGGCATAGGTCACAAC
TBE9439s	CCCGIGATGGAGGCTGCATCA
cFD3*	AGCATGTCTTCCGTGGTCATCCA
TBE10041s	CGCACGACSTGGAGCATTCAT
TBE10687a	CGCTGCCGCAKCATTCCAT
TBE10430s	TGGAGTGCTCGTTAAATATTGT
TBE11117a	AGCGGGTGTTTTTCCGAGTCACACA

S2 Table: Primers used for sequencing. * primers adopted from [38]
