# Supplementary material for: The Baikal subtype of tick-borne encephalitis virus is evident of recombination between Siberian and Far-Eastern subtypes
Source: PLoS Negl Trop Dis. 2023 Mar 27;17(3):e0011141. doi: 10.1371/journal.pntd.0011141 (PMC10079218; doi:10.1371/journal.pntd.0011141)
Supplement: S3 Table — (PDF) [file pntd.0011141.s003.pdf]

		JCF1			JCF2			JCF3		
node name	Node on the tree	median height, years ago	95% HPD of median height	posterior probability	median height, years ago	95% HPD of median height	posterior probability	median height, years ago	95% HPD of median height	posterior probability
tree root	**A**	4376.2	2478.0-6357.6	1	3731	565.9-7546.9	1	4546.4	2590.0-6470.2	1
Siberian subtype and Far-Eastern subtype MRCA	**B**	984	381.0-1778.0	0.93	1433.6	209.7-4215.1	1	675.5	229.8-1351.0	0.48
Baikal subtype and Far-Eastern subtype MRCA	**C**	688.3	257.3-1272.1	1	---			---		
Baikal subtype and Siberian subtype MRCA	**D**	---			973	158.0-3027.5	1	---		
Siberian subtype MRCA	**E**	479.1	190.5-875.1	1	682	119.4-1726.8	1	423.8	135.6-864.8	0.97
Far-Eastern subtype MRCA	**F**	381.9	156.9-687.8	1	482.9	71.4-1170.1	1	---	---	---
Baikal subtype and Far-Eastern subtype MRCA	**J**	---			---			435.9	151.4-853.7	1
Shenjang lineage of Far-Eastern subtype and Baikal subtype MRCA	**G**	---			---			293	101.7-592.4	0.61
Baikal subtype within Shenjang lineage of Far-Eastern subtype	**H**	---			---			235	81.3-491.7	0.77
Baikal subtype MRCA	**I**	90	25.0-198.7	1	119	14.2-434.8	1	83.2	23.1-187.6	1

S3 Table: Heights and posterior probabilities of select nodes shown in S2 Fig from the reconstructed Bayesian phylogeny of TBEV JCFs.
