# Supplementary figures and images for: The Baikal subtype of tick-borne encephalitis virus is evident of recombination between Siberian and Far-Eastern subtypes
Source: PLoS Negl Trop Dis. 2023 Mar 27;17(3):e0011141. doi: 10.1371/journal.pntd.0011141 (PMC10079218; doi:10.1371/journal.pntd.0011141)

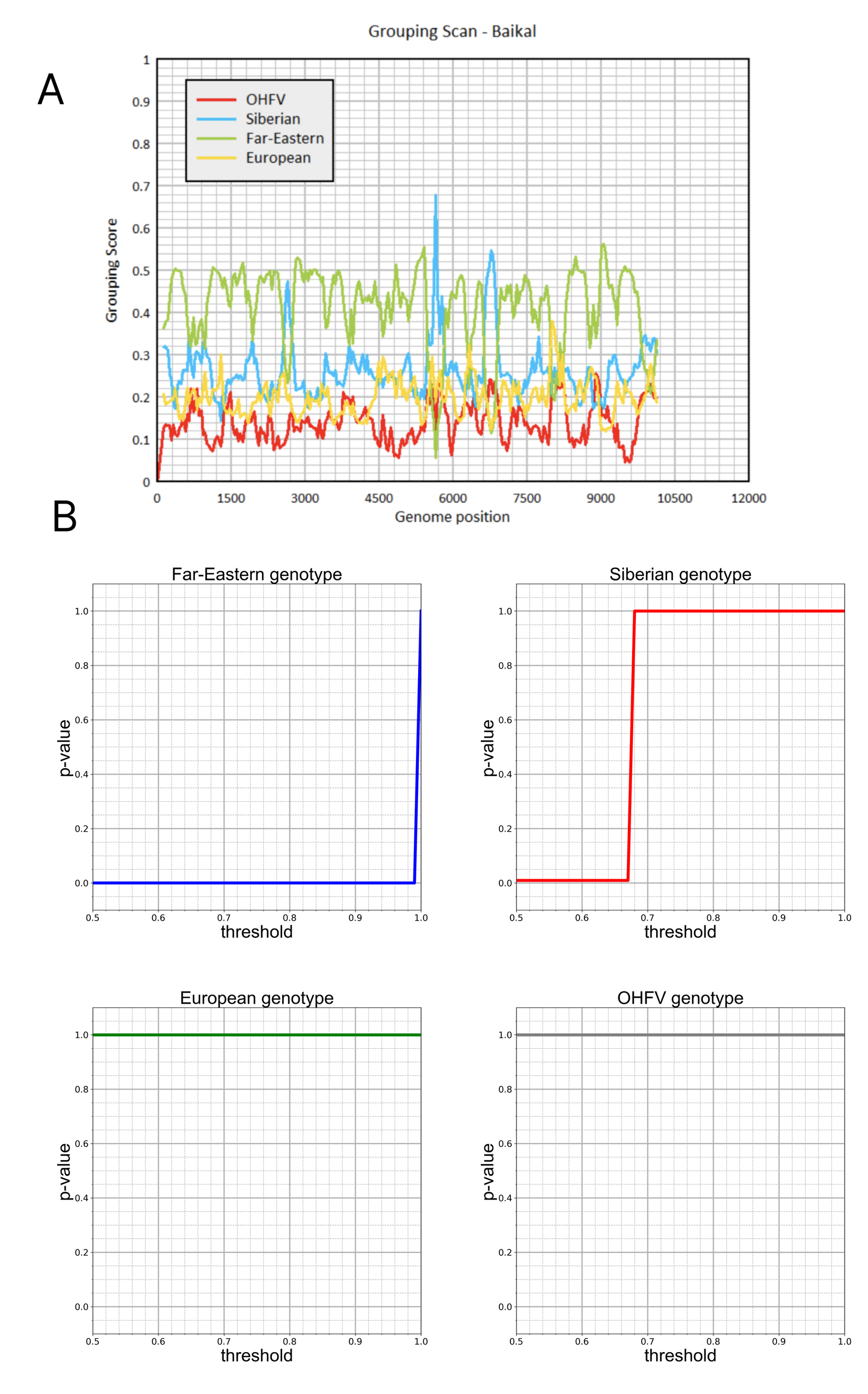

Supplement: S1 Fig — (A) GS analysis of the artificially generated alignment with recombination; (B) GSAUC curves corresponding to the GS analysis. (TIF) [file pntd.0011141.s004.tif]

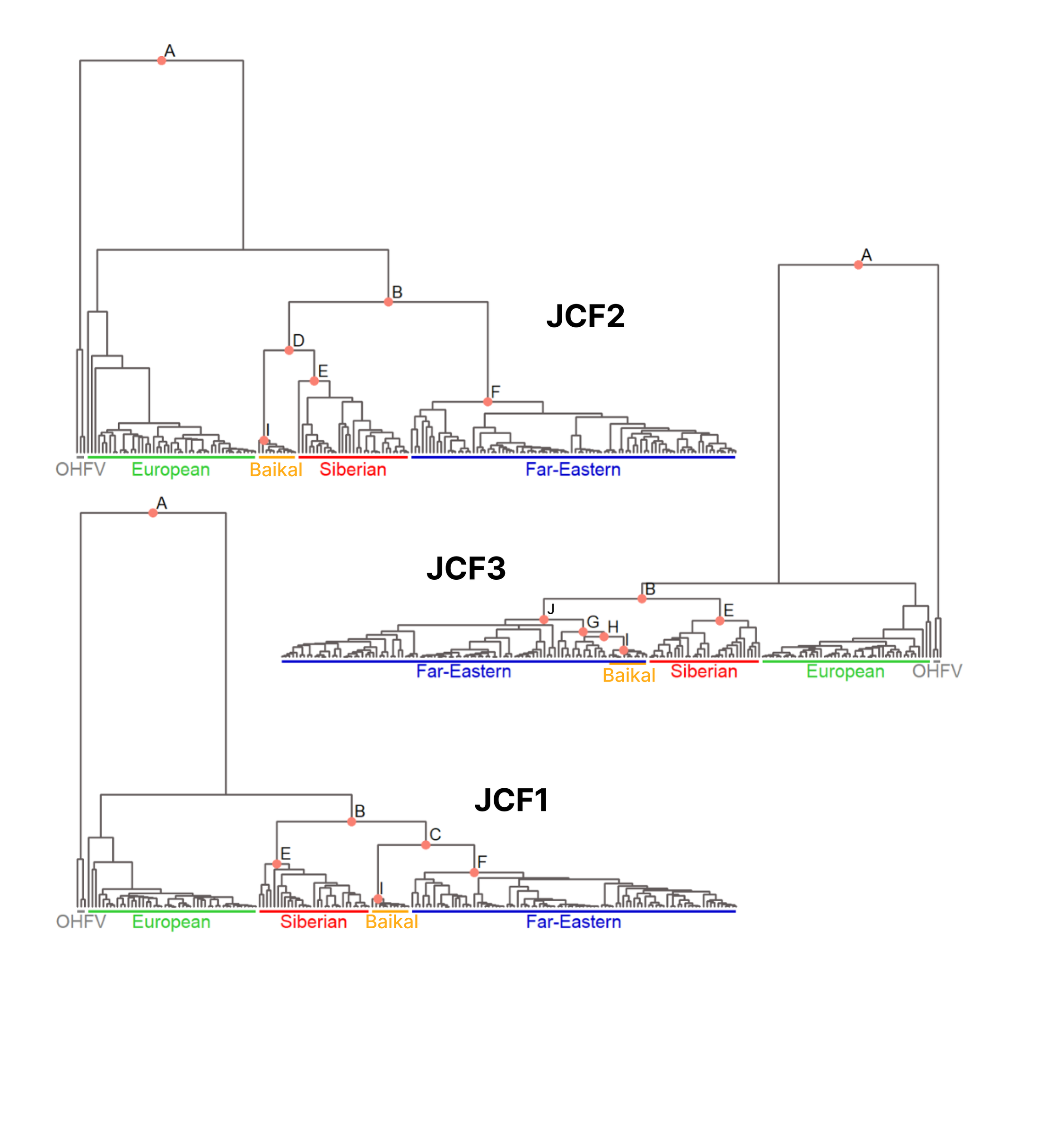

Supplement: S2 Fig — (TIF) [file pntd.0011141.s005.tif]
