# Supplementary material for: The Baikal subtype of tick-borne encephalitis virus is evident of recombination between Siberian and Far-Eastern subtypes
Source: PLoS Negl Trop Dis. 2023 Mar 27;17(3):e0011141. doi: 10.1371/journal.pntd.0011141 (PMC10079218; doi:10.1371/journal.pntd.0011141)
Supplement: Attachment — Submitted filename: MinorRevision_ReviewerComments_20220706.docx [file pntd.0011141.s006.docx]

**Title: “The recently identified long-living lineage of the tick-borne encephalitis virus is a result of an ancient recombination event”**

**Authors:** Sukhorukov GA, Paramonov AI, Lisak OV, Kozlova IV, Bazykin GA, *et al*.

**Comments to the Authors:**

In this manuscript, Sukhorukov *et al*. introduce a newly developed method to infer ancestral recombination events in the tick-borne encephalitis virus (TBEV) genome. Here, the authors implemented a recombination model (*i.e.*, GSAUC method) to specifically detect and validate evolutionary signals of recombination events within the TBEV 886-84-like lineage; in particular, the evidence for two proposed recombination signatures were outlined between the ancestral Far Eastern (FE) and Siberian genotypes and within the more recent Far Eastern Shenjang lineage and 886-84-like clade.

This document is well-written. In particular, the implemented GSAUC method computational model, hypothesis testing, and data interpretation/validation are all outlined in an intuitive framework which will allow readers to reproduce or translate the proposed method via the provided GitHub link and referenced software packages. In preparation, Sukhorukov *et al*. could consider further validation of the robustness of their testing algorithm using engineered *in silico* data models or additional, comparative software packages/code repositories.

I recommend this manuscript for publication in *PLOS Neglected Tropical Diseases* pending review of the provided revisions and suggestions/considerations (see below).

**Revisions:**

**Pg. 4 Par. 2 Line 17:** Provide the expanded definition for “AUC” in the “GSAUC” acronym.

**Pg. 6 Par. 2 Line 1:** How did the authors determine which model (*i.e.*, Kimura’s 2-parameter model) best fit the applied TBEV sequence alignment?

**Pg. 13 Par. 3:** Several indicated node and LCA values for the JCF1 and JCF2 phylogenies (*e.g.*, nodes C and D) are inconsistent with the referenced supplemental Figure S1 infographics.

**Figure 1:** Consider editing the applied color-scheme for the bootstrap legend to allow better visibility/step differentiation (such as an orange-blue scale). Currently, it is difficult (partially due to size) to discriminate between the green lines (0.95 and 1.00) and find the bright yellow (0.90) colored lines.

**Figure S1:** Based on the JCF2 phylogeny, the associated metadata for Node C appears to be incorrectly listed in the JCF1 versus intended JCF2 column. In addition, similar metadata for Node D is listed in the JCF2 column despite annotation on the JCF1 phylogeny. Relative to Figure 5, these discrepancies may stem from the apparent swap of the labeled JCF1 and JCF2 phylogenies in Figure S1.

**Comments & Suggestions:**

**Pg. 9 Par. 2 Line 3-5 (and Figure 2):** Expand upon the provided text to clarify how the results of the applied RDP4 package analytics: TOPAL, SIMPLOT, and BootScan support signatures of Far Eastern and Siberian genotype recombination. Inclusion of these tests provides an analytical foundation the authors build upon in the proposed GSAUC method, and readers may be unfamiliar with interpretation of these results. In brief, what particular features of the TOPAL, SIMPLOT, and BootScan output support (or potentially contradict) the results of the applied GSAUC method? Minimally, expansion of the descriptive text in the Figure 2 legend would be highly informative (such as the labeled thresholds and gray-scaled line series in the TOPAL/DSS score panel).

**Methods:** Inclusion of an additional positive control or engineered *in silico* model for TBEV recombination (similar to the cited Bertrand *et al*. reference) may further strengthen the robustness of the proposed GSAUC method. Does this method detect artificially-introduced recombination events? Furthermore, how do the alternative RDP4 methodologies perform as a baseline in these same scenarios?

**Discussion:** Sukhorukov *et al.* might consider addressing how this new methodology can be applied more broadly in their field. Is the GSAUC method limited to the detection of ancestral recombination events (due to underlying model assumptions/parameters), or can this tool be applied to more recent evolutionary scenarios in other viral systems? Inclusion of additional text would highlight inappropriate applications and strengthen readership impact/utility.

**Figure/Table Descriptions:** In general, provision of more descriptive text for applied data series, axes, thresholds, color-schemes, icons, and numerical labels will aid in orienting readers and data interpretation.

**Figure 3**: Based on the provided inclusion criteria and the results detailed in Figures 3 and 4, GS scores for the Siberian genotype exceeded the defined 0.5 threshold with GSAUC statistic p-values < 0.05 in the encoded capsid and prM-E gene segments. Why did the authors disqualify these signals as putative joint characteristic fragments (JCFs)?
