# Supplementary material for: The Baikal subtype of tick-borne encephalitis virus is evident of recombination between Siberian and Far-Eastern subtypes
Source: PLoS Negl Trop Dis. 2023 Mar 27;17(3):e0011141. doi: 10.1371/journal.pntd.0011141 (PMC10079218; doi:10.1371/journal.pntd.0011141)
Supplement: Attachment — Submitted filename: review_answer_PLOS.docx [file pntd.0011141.s007.docx]

We thank all reviewers for their extensive comments which allowed us to improve the manuscript.

# Reviewer 1

## New analyses required for acceptance

Revisions …

Pg. 6 Par. 2 Line 1: How did the authors determine which model (i.e., Kimura’s 2-parameter model) best fit the applied TBEV sequence alignment?

To formalize model choice, we now run the ModelFinder in IQTree2 for each of the 10 random fragments of TBEV sequence alignment, each 250 nucleotides long, matching the window length of GS; as well as the JCF2 and JCF3 fragments. In all 12 tests, the best-fitting model was one of the following: TN, TIM2, TIM3, or GTR. All these models involve more parameters than the most parameter-rich model implemented in GS, i.e., K80, making K80 our model of choice. This is now described in Methods.

Comments & Suggestions ...

Methods: Inclusion of an additional positive control or engineered in silico model for TBEV recombination (similar to the cited Bertrand et al. reference) may further strengthen the robustness of the proposed GSAUC method. Does this method detect artificially-introduced recombination events? Furthermore, how do the alternative RDP4 methodologies perform as a baseline in these same scenarios?

As suggested by the Reviewer, we now generate an in silico recombination with the characteristics matching those of the proposed Far Eastern - Siberian recombination at the origin of the Baikal clade, and test GSAUC on this positive control. The simulated recombination is detected by GSAUC reliably:

|  |  |
| --- | --- |
|  |  |

We now include this analysis in the text (Fig S1).

The in silico generated recombination is also detected by BootScan. However, this signal is weaker, and also a spurious high score for OHFV is observed:

## Results

Revisions ...

Pg. 13 Par. 3: Several indicated node and LCA values for the JCF1 and JCF2 phylogenies (e.g., nodes C and D) are inconsistent with the referenced supplemental Figure S1 infographics.

Thank you for spotting this error in tree annotation, which affects this and the next comment; now fixed.

Figure S1: Based on the JCF2 phylogeny, the associated metadata for Node C appears to be incorrectly listed in the JCF1 versus intended JCF2 column. In addition, similar metadata for Node D is listed in the JCF2 column despite annotation on the JCF1 phylogeny. Relative to Figure 5, these discrepancies may stem from the apparent swap of the labeled JCF1 and JCF2 phylogenies in Figure S1.

This resulted from the same error as the previous comment; also fixed, thank you.

Comments & Suggestions ...

Pg. 9 Par. 2 Line 3-5 (and Figure 2): Expand upon the provided text to clarify how the results of the applied RDP4 package analytics: TOPAL, SIMPLOT, and BootScan support signatures of Far Eastern and Siberian genotype recombination. Inclusion of these tests provides an analytical foundation the authors build upon in the proposed GSAUC method, and readers may be unfamiliar with interpretation of these results. In brief, what particular features of the TOPAL, SIMPLOT, and BootScan output support (or potentially contradict) the results of the applied GSAUC method? Minimally, expansion of the descriptive text in the Figure 2 legend would be highly informative (such as the labeled thresholds and gray-scaled line series in the TOPAL/DSS score panel).

As suggested by the Reviewer, we now provide interpretation of results of the RDP4 analyses in Figure 2 caption and in para 2 of the Results section. We also combine figures 2 and 3 in the new Figure 2, hopefully simplifying comparisons of results of different analyses and streamlining our narrative.

Figure 3: Based on the provided inclusion criteria and the results detailed in Figures 3 and 4, GS scores for the Siberian genotype exceeded the defined 0.5 threshold with GSAUC statistic p-values < 0.05 in the encoded capsid and prM-E gene segments. Why did the authors disqualify these signals as putative joint characteristic fragments (JCFs)?

We now state our criteria for choice of JCFs more formally. We reasoned that a single window might exceed the 0.5 threshold due to random phylogenetic noise, but this is less likely for multiple adjacent windows. Therefore, for a fragment to be picked as a part of a JCF, we required it to exceed the 0.5 threshold in at least two adjacent windows. There were exactly three such segments in our alignment: the two segments comprising JFC2 (with respectively two and three adjacent windows exceeding the threshold), and the single segment comprising JCF3 (with 8 such adjacent windows; Figure 2D). This is now explained in Methods.

## Conclusions

Comments & Suggestions ...

Discussion: Sukhorukov et al. might consider addressing how this new methodology can be applied more broadly in their field. Is the GSAUC method limited to the detection of ancestral recombination events (due to underlying model assumptions/parameters), or can this tool be applied to more recent evolutionary scenarios in other viral systems? Inclusion of additional text would highlight inappropriate applications and strengthen readership impact/utility.

In the revision, we now include an in silico generated recombination with characteristics matching those of the proposed Siberian - Far Eastern recombination, and show that GSAUC performs robustly under these parameters. We also now discuss why it is easier to detect recombination between more distant strains in the beginning of Discussion, and mention that GSAUC, similar to other methods, is expected to perform better in such a scenario. Overall, we expect GSAUC to perform well for detection of recombination at origin of robust clades consisting of multiple samples.

## Editorial and Data Presentation Modifications

Revisions ...

Pg. 4 Par. 2 Line 17: Provide the expanded definition for “AUC” in the “GSAUC” acronym.

This acronym is defined below (p. 7); for clarity, we now remove all mention of GSAUC before p. 7.

Figure 1: Consider editing the applied color-scheme for the bootstrap legend to allow better visibility/step differentiation (such as an orange-blue scale). Currently, it is difficult (partially due to size) to discriminate between the green lines (0.95 and 1.00) and find the bright yellow (0.90) colored lines.

Color coding now replaced with circle sizes for better visibility.

Comments & Suggestions ...

Figure/Table Descriptions: In general, provision of more descriptive text for applied data series, axes, thresholds, color-schemes, icons, and numerical labels will aid in orienting readers and data interpretation.

We now revised the figure and table captions for clarity and completeness.

## Summary and General Comments

In this manuscript, Sukhorukov et al. introduce a newly developed method to infer ancestral recombination events in the tick-borne encephalitis virus (TBEV) genome. Here, the authors implemented a recombination model (i.e., GSAUC method) to specifically detect and validate evolutionary signals of recombination events within the TBEV 886-84-like lineage; in particular, the evidence for two proposed recombination signatures were outlined between the ancestral Far Eastern (FE) and Siberian genotypes and within the more recent Far Eastern Shenjang lineage and 886-84-like clade.

This document is well-written. In particular, the implemented GSAUC method computational model, hypothesis testing, and data interpretation/validation are all outlined in an intuitive framework which will allow readers to reproduce or translate the proposed method via the provided GitHub link and referenced software packages. In preparation, Sukhorukov et al. could consider further validation of the robustness of their testing algorithm using engineered in silico data models or additional, comparative software packages/code repositories.

We thank the Reviewer for the positive comments.

# Reviewer 2

## New analyses required for acceptance

The authors excluded two most divergent variants of the Siberian and Far-East genotypes (TBEV-178-79 (GenBank ID EF469661) and TBEV-2871 (GenBank ID MF774565)) from the presented analysis. Why not improve the power of the analysis, given the otherwise limited virus sampling and low genomic variation that hindered prior analyses? If these viruses are indeed recombinants, as the authors concern, any evidence in support of this assertion will strengthen the main conclusion of the study. If the inclusion of these two sequences affects the main conclusion of this study, this result could be used to reveal critical dependencies of the recombination analysis and call for further expansion of genomic exploration of the TBEV natural diversity. One way or another, confidence of conclusions, original or revised, would be improved.

In our analysis, we focused on the well-established clades supported by multiple samples. By contrast, both these sequences, TBEV-178-79 and TBEV-2871, are sole representatives of their clades, making them more dubious. Aside from additional recombination events, they could result from e.g. cross-contamination.

Still, below, we provide the results of the GS analysis using these two samples, TBEV-178-79 (left) and TBEV-2871 (right), as a query. For both sequences, we observe putative evidence for recombination at their origin. If additional representatives of these clades are described in the future, they can be tested for recombination using the methods we describe here.

|  |  |
| --- | --- |

As proposed by the Reviewer, we also tested if inclusion of these two sequences affects the results of the GS analysis for the Baikal clade. We find that our main results - evidence for recombination involving the Siberian and Far-Eastern clades - hold: similar to the main analysis, these clades still have the highest GS scores around positions 6750 and 8000 of the alignment, respectively. The high scores observed for 178-79 at much of the alignment simply indicate that this is a sister clade (there is no way for a query sequence to be “embedded” in a clade consisting of just one sample).

- Consensus sequence versus all sequences. The authors used consensus sequences of genotypes (groups) for recombination analysis without providing a rational and acknowledging that it may decrease the analysis power. Besides, there are two other issues with this choice: a) no details provided about how consensus sequences were generated, including the use of weighting or another method to correct for uneven virus sampling (even if the two most divergent sequences were excluded); b) despite consensus sequence being used for each group, GS score calculation relies on analysis of all sequences in each group (p. 6), which is confusing.

The three RDP4 methods that we use as the baseline, TOPAL/DSS, SIMPLOT and Bootscan, all by their design only take individual representatives of each parental lineage as input, and therefore there is no way to provide them with anything other than the consensus. By contrast, the newly developed GS analysis indeed analyzes all sequences in parental groups, which we consider one of its advantages. This is now clarified. The simplest method for consensus reconstruction was used, i.e., majority rule and no weighting (now mentioned). This is in line with the usual practice, and we are unsure what the motivation for weighting could be.

- Provide a Supplementary Table detailing sequences used in the study

The sequence IDs and the sampling details for the four sequences obtained in this study are provided in Table S1. The sequence IDs for all sequences used, including those obtained by others, and their alignment are provided in GitHub (https://github.com/gregoruar/tbev_rec/blob/master/full_alignment.fasta).

## Results

Provide a chapter describing four new TBEVs and their genome sequences and relationship with known TBEVs. Acknowledge the mouse and tissue culture propagation of TBEV as sources of extra mutations. Its (limited?) scale and impact on downstream inferences should be discussed in the Discussion section.

The sampling, isolation, culturing, genome amplification and sequencing of the four new TBEVs are provided in the first two chapters of Materials and Methods. The raw phylogenetic relationships with known TBEVs are shown in Fig. 1. We now mention the mouse and tissue culture propagation of TBEV in the first chapter of Results, and discuss that they are unlikely to affect our analyses.

- p. 9: detail results presented in Fig. 2, e,g. plot distribution, peaks, agreement between different methods, type of evidence, etc.

We now provide as expanded Figure 2 caption detailing these analyses, and include details in the text.

- the time-based analysis should be moved from Discussion p.13 to the Results if retained in the paper at all, which is uncertain. The lack of the root-to-tip regression signal and high uncertainty of the HPD ranges (see below) question the reliability of this analysis. Choice of the outgroup and testing of evolutionary models should be substantiated.

We include a paragraph of disclaimers prior to presenting the time-based analysis, hopefully making our point that this analysis should only be considered preliminary. For the same reason, we would rather keep it in Discussion rather than move it to Results. However, we believe that it is helpful to provide a suggestion for a rough order of events. We provide the HPD ranges for all estimates, indicating their high uncertainty.

To address the comment on testing of evolutionary models, we now perform a formal model selection for BEAST analysis using IQtree. The best-fit model turns out to be GTR+F+I+G4 model for JCF1, TIM2e+G4 for JCF2, and TIM+F+I+G4 for JCF3. For all these models, the closest model among those implemented in BEAST is GTR+Gamma, the model we use. This analysis is now included in Methods.

The fact that the Omsk Hemorrhagic Fever virus (OHFV) is an outgroup to all TBEV strains is a result of previous studies (Walker, 2022). We now mention this. Notably, the GS analysis of recombination uses unrooted trees so our main results are independent of outgroup choice.

*P. J. Walker et al., “Recent changes to virus taxonomy ratified by the International Committee on Taxonomy of Viruses (2022),” Arch. Virol., Aug. 2022, doi: 10.1007/s00705-022-05516-5.*

- Move Fig. 3 GS and genomic plots to the bottom and top of Fig. 2, respectively. This will facilitate comparison and reveal agreement between results of different methods.

Thank you for this suggestion. We now combine Figs. 2 and 3 in the new Fig. 2.

- Figure 4: explain why plots look continuous rather than discrete functions, despite the underlying analysis uses a sliding window with a discrete step.

We used a small discrete step for thresholds (0.01), making the plots look smooth. This is now mentioned in the figure caption.

Could the function be extended below 0.5? Could a log scale be used at the Y axis and the p-value cut-offs of 0.05 and 0.01 be specified ?

While it is possible to extend the plots to threshold values below 0.5, this makes no biological sense as values below 0.5 indicate that the query clade actually is remote from the target clade. We cannot use the log scale as p=0 for some thresholds.

- What is a tree topology of phylogeny that is based on the entire genome minus JCF2 and JCF3? Does it match the topology of Fig. 1 and JCF1 trees? If so, why not use it instead of JCF1, which selection is a bit arbitrary?

Yes, the topology of the tree of the entire genome minus JCF2 and JCF3 is the same as that of the JCF1 tree - see the figure below. For the main text, we use the shorter region JCF1 to eliminate the concern that the differences in topologies are somehow caused by differences in genome lengths, e.g. due to evolution rate heterogeneity in a larger sequence segment.

- Provide sequence alignment(s) to illustrate and support recombination

The alignments are now available on GitHub:

https://github.com/gregoruar/tbev_rec/blob/master/full_alignment.fasta

## Conclusions

See Methods

- The manuscript is dominated by the use of “recombination” without acknowledgement that obtained results are about the incongruence of trees for different genomic regions, with a homologous recombination being one of possible explanations of the observed patterns. Accordingly, the word “recombination” should be used carefully and seldom, and other explanations for the obtained results should be presented and, if discarded, explicit reasoning should be provided. - The same is implied to the title and conclusions.

We now explicitly state that the evidence for recombination comes from incongruence of trees for different genomic regions, including in the Abstract and in the newly added limitations paragraph of the Discussion. We discuss the alternative explanations, including convergence caused by natural selection or biased mutagenesis.

- Limitations of the study are yet to be specified

We now include an explicit paragraph on limitations in the Discussion. We also expand the limitations of the temporal analysis.

## Editorial and Data Presentation Modifications

Introduction.

-	Note that most analysed TBEV sequences are just sequences, they are “variants” but not “strains”, that designation is reserved for well characterized variants.

We now replace all instances of “strains” with “variants” as suggested.

-	Up-to-date taxonomy of the Flaviviridae including how it must be spelled and written is here (10.1099/jgv.0.000672)

We were unable to spot any errors in naming of taxa. In the text, we use the names *Flaviviridae* for the family, and *Flavivirus* (Latin) or flavivirus (English) for the genus, in line with the reference proposed by the Reviewer.

-	The presented analysis concerns a flavivirus species, which includes all variants of TBEV and this species should be specified. Consult https://doi.org/10.1371/journal.ppat.1009318 for understanding differences between viruses and virus species.

We now refer to TBEV as a viral species in the first phrase of the introduction.

-	TBEV genome is mRNA that is translated to produce polyprotein, cleaved by virus and host proteases to mature proteins.

We revised the corresponding phrase in the Introduction.

-	Describe the basis of the TBEV genotyping, e.g. genome region, method, criterion etc

A fragment of E protein coding sequence is commonly used for TBEV subtyping. The E gene 10% nucleotide sequence distance threshold clearly supported the segregation of seven TBEV subtypes: TBEV-Eu, TBEV-Sib, TBEV-FE, TBEV-Ob (TBEV-2871), TBEV-Him, TBEV-Bkl-1 (TBEV-178-79), and TBEV-Bkl-2 (TBEV-886-84) (Deviatkin AA, Karganova GG, Vakulenko YA, Lukashev AN. TBEV Subtyping in Terms of Genetic Distance. Viruses. 2020 Oct 31;12(11):1240. doi: 10.3390/v12111240).

-	Describe evidence for homologous recombination in TBEV (genome regions, genotypes) and causes of uncertainty of these findings.

We provide the following evidence in the text:

1) Results of the 3 recombination analyses from RDP4;
2) Results of the GS analysis, with statistical significance assessed using the GSAUC p-value calculation;
3) The reconstructed Bayesian phylogeny for the regions with the strongest signal.

All these points are covered in detail in Results and Discussion.

We now also include a paragraph on the limitation of study in the Discussion.

-	p.3. Provide reference for the observation of infection by multiple TBEV genotypes of a patient.

References added (Karan et al. 2007; Pogodina et al. 2007; Pogodina et al. 2012).

-	“In the agar…” sentence does not have sense

Rephrased.

Discussion

-	p.11: discuss “evidence for it [recombination] has been controversial”. Show how the controversial points were addressed in this study, especially a role of the new statistics; why it is superior compared for the Topal statistic, for instance.

The previous reports were controversial because the 1st report was found to be a lab contamination, and the 2nd report was disproved by use of different statistics. This is explained in paragraph 3 of Introduction.

A number of points make our report more convincing than the previous ones: (i) the fact that recombination is described for a phylogenetically established cluster (>10 sequences), rather than an individual variant; (ii) use of a range of recombination detection methods that all converge; (iii) reconstructed bayesian phylogeny with very high posterior values that conflicted between genomic regions. The role of the new statistic is to provide a way to formally assess the significance of the GS results.

-	p.11: make explicit reference to tree nodes (A, B, C, etc) in Fig. S1, when discussing the results.

Added as suggested.

-	p.12: based on the Results section, it is misstatement to list the JCF1 region as of the possible recombinant origin; should be corrected

Corrected to “regions with different evolutionary histories”

Others:

-	Specify genome fractions occupied by JCF2 and JCF3

Added as suggested.

-	Could you say that JCF2 and JCF3 are the only regions of possible recombinant origins, given the analyzed dataset?

These are the two regions for which we have (we believe) robust evidence, including statistical support. However, we cannot exclude additional recombination events in the history of these variants, especially if such events affected closely related variants..

-	Illustrations and elsewhere: make clear difference between TBEV genotypes of the same virus species and the outgroup, which belongs to another flavivirus species.

We now specify throughout the text and figure captions that OHFV is a different species used as outgroup.

-	Figures 2-4: specify query

We now specify that the 886-84-like lineage was used as the query in the caption of Figure 2, which now also includes Figure 3 of the previous version. We do not refer to the query in Figure 4 caption (now Figure 3 caption).

-	Fig. S1: a) Results for JCF1 and JCF2 may have been swapped relative to Fig. 5 and Fig. S1-associated Table;

Now fixed, thank you.

b) 95% HPD for many nodes are highly uncertain that need to be discussed in respect to the time-based inferences;

We now include this statement in the Discussion, together with an overall discussion of the robustness of the temporal signal in this data.

c) some nodes for three phylogenies must have different names (e.g. F node for JCF3 versus JCF1 and JCF2)

Indeed, the position of the “F” node was ambiguous between the panels; now fixed. There was just one such node.

## Summary and General Comments

Sukhorukov et al., present new genome sequences of four TBEV collected in the Baikal area of Russia over six years and phylogenomic analysis of TBEV sequences available on 2018 along with the newly sequenced genomes. Using diverse software and an original statistical test, developed for this study, they found support for conflicting evolutionary histories of different genome regions encoded by a TBEV group known as 886-84-like cluster (genotype), including the new genome sequences. The authors proposed that an ancestor of this cluster emerged as result of ancient recombination(s) between ancestors of two main TBEV genotypes, Siberian and Far-East.

# Reviewer 3

## New analyses required for acceptance

Objectives of the study are clearly articulated, the design is appropriate.

## Results

The results are clearly and completely presented.

## Conclusions

The conclusions are correct and supported by the data presented.

## Editorial and Data Presentation Modifications

Specific comments:

1. Title: It would be good to change the title to reflect better the obtained results. For example, the Baikal TBEV genotype is a result of a recombination between Siberan and Far-Eastern TBEV strains.

We thank the Reviewer for this suggestion of a title, which we gratefully use.

2. Abstract: The Baikal and Himalayan genotypes are already considered as new TBEV genotypes.

We now change “886-84-like” to “”Baikal” throughout the manuscript.

3. Abstract: TBEV samples – better: TBEV strains

Reviewer 2 discouraged the use of “strains”; changed to “variants” instead.

4. Introduction: Flavivirus genus is not the only genus of the family Flaviviridae

Thank you, corrected.

5. The reference number 1 is not appropriate. Please, cite any recent review or ICTV release.

Changed as suggested.

6. “short single-stranded positive-sense RNA viruses” – unusual wording

Revised for clarity.

7. “encodes a single transcript that is translated” – better: encodes a single open reading frame that is translated

Revised in line with this suggestion and that of Reviewer 2.

8. it is not clear how the non-structural proteins are involved in transmission of the virus?

This phrase is now removed.

9. Introduction second paragraph: Far East (capital F)

We meant that its range stretches all the way to Eastern Siberia; now rephrased.

10. Page 3 and further: in the scientific literature a name “Baikalian subtype/genotype” is already well established instead of “886-84-like”. The “886-84-like” name is not well known in the Western literature and most papers use just the name “Baikalian”. This reviewer strongly encourages to use the name Baikalian genotype thorough the manuscript.

We now change “886-84-like” to “”Baikal” throughout (“Baikal” rather than “Baikalian” because this is the original and more frequently used name).

11. Page 3 end of the second paragraph: a reference is missing

Thank you, corrected.

12. Page 3 third paragraph: not “reaction of neutralization” but “neutralization test” or “neutralization assay”

Changed.

13. Page 4 second paragraph: … study, we developed….

Changed as suggested.

14. Figure 2: please, prepare a better figure legend. It is not good to say only “results from the analysis”. The legend should be self-explanatory; i.e., describe what was the purpose, what was done and what is shown. The same for Figure 2 and 5.

We have now expanded radically these legends, explaining the analyses and interpretation.

## Summary and General Comments

Sukhorukov et al. submitted a manuscript titled “The recently identified long-living lineage of the tick-borne encephalitis virus is a result of an ancient recombination event” for peer-review procedure in PLoS Neglected Tropical Diseases. The manuscript identified that the newly described Baikalian TBEV subtype may be a result or a recombination event between Siberian and Far-Eastern TBEV strains. This discovery is of interest and provides the first clear and statistically solid evidence of recombination in TBEV. However, the title and several other issues listed above need to be addressed before the manuscript can be considered acceptable for publication.
