# Supplementary material for: The Baikal subtype of tick-borne encephalitis virus is evident of recombination between Siberian and Far-Eastern subtypes
Source: PLoS Negl Trop Dis. 2023 Mar 27;17(3):e0011141. doi: 10.1371/journal.pntd.0011141 (PMC10079218; doi:10.1371/journal.pntd.0011141)
Supplement: Attachment — Submitted filename: review_answer_2.docx [file pntd.0011141.s008.docx]

We thank all reviewers for their feedback and careful reading of the manuscript.

Reviewer's Responses to Questions

Key Review Criteria Required for Acceptance?

Reviewer #1: In this revised manuscript, Sukhorukov et al. comprehensively address all provided reviewer comments in addition to suggestions for further supporting evidence/analytics. In particular, expansion of the methods section and figure/table caption text will help orient and guide readers through the logic of the presented data analyses.

Reviewer #3: (No Response)

--------------------

Results

Reviewer #1: Minor Revisions:

Pg. 10 Par. 3 Title: (grammatical) Alternatively, consider “The Baikal subtype is evidence of recombination.”

Changed to “evident of recombination”

Pg. 13 Par. 4 Line 3: Replace the reference to “node F” with “node J.” There is no node F in the revised JCF3 phylogeny (see additional Figure S2 table revision below).

Thanks, it was an error, we modified the corresponding table in FigureS2 and the text accordingly.

Reviewer #3: (No Response)

--------------------

Conclusions

Reviewer #1: Minor Revisions:

Pg. 15 Par. 2 Line 1: How do the mentioned Figure 3 plots indicate putative recombination segment length? Do the authors mean to reference Figure 2D instead where JCF1, JCF2, and JCF3 are annotated relative to genetic position?

Thanks for noticing this error, the reference should have been to Fig. 2D. Now corrected.

Pg. 16 Par 2 Line 2: No LCA value of 678 is provided in the Figure S2 table. Is the value 676 (rounded from 675.5) for the respective node B median height intended?

Again, thanks for spotting this. The correct value is 676, now corrected.

Reviewer #3: (No Response)

--------------------

Editorial and Data Presentation Modifications?

Reviewer #1: Minor Revisions:

Pg. 4 Par. 3 Line 2: (grammatical) Consider “Most of these methods evaluate the strength...” instead.

Changed as suggested.

Table S1: How do three asterisks differ or encapsulate the provided one and two asterisk descriptions? Alternatively, consider use of numerical superscript annotation (e.g., 1, 2, 3, etc.) instead.

The notation in table is now revised for clarity. We now use asterisk to show the sequenced samples, and put the information on the tissue in the table itself.

Figure S2: Replace all “886-84-like” references with “Baikal subtype” to be consistent with the rest of the manuscript (if applicable).

Now corrected.

Figure S2 (table): Remove the entry for node F in the JCF3 column. This information appears to have been moved to node J in this column based on the revised supplemental figure.

Now corrected.

Comments & Suggestions:

Figures: Streamline the subtype-specific color-schemes between all figures. For example, the colors applied in Figure S1A do not match those applied in all the other figures.

Coloring scheme in Figure S1A now matches that in the rest of the manuscript.

Figure 2 Description: Provide more specific text than “in some of the alignment regions” or “for some regions” where indicated. For example, the authors may consider highlighting how indicated Joint characteristic fragments (JCFs) annotated in the GS analysis (2D) correspond to positional segments which demonstrate graphical shifts in genetic distance (2B) and/or bootstrap support (2C) to specific subtypes as compared to surrounding positional segments.

Revised as suggested.

--------------------

Summary and General Comments

Reviewer #1: No additional major revisions or new analyses are recommended. Provided revisions and comments are minor with a few data/graphical inconsistencies indicated within the manuscript text and figures/tables.

Reviewer #3: The authors addressed all my comments. I have no further comments on this submission.
